# Acute ST-Elevation Myocardial Infarction Complicating Infective Endocarditis: A Case Report and Review of Reported Outcomes After Revascularization

**DOI:** 10.7759/cureus.104883

**Published:** 2026-03-09

**Authors:** Hatim Al Lawati, Mohamed M Madan, Ahmed Shams

**Affiliations:** 1 Adult Cardiology, The National Heart Centre, The Royal Hospital, Muscat, OMN; 2 Medicine, College of Medicine and Health Sciences, National University of Science and Technology, Sohar, OMN

**Keywords:** aortic endocarditis, coronary artery bypass grafting (cabg), infective endocarditis, primary pci, septic embolism, stemi (st elevation myocardial infarction)

## Abstract

Acute myocardial infarction in the context of infective endocarditis is a dreadful complication. Various mechanisms have been proposed to explain this complication; embolization of fragments of valvular vegetations into the coronary tree is one possible mechanism. Early mortality with this complication is reportedly high. Moreover, outcomes after treatment, either with percutaneous or surgical intervention, are not well-described in the literature. We report a case of acute ST-elevation myocardial infarction complicating bacterial native aortic valve endocarditis. Despite aspiration of infective debris and stent implantation in the left anterior descending coronary artery, the patient died from cardiogenic and septic shock. We also summarize the literature reporting outcomes of such patients undergoing percutaneous coronary intervention and comparing them to the outcomes of patients undergoing surgical revascularization.

## Introduction

Acute myocardial infarction (AMI) complicating infective endocarditis (IE) represents a unique clinical challenge. It can be the manifestation at presentation or develop at any stage of the disease, either before treatment or even several weeks into culture-guided antimicrobial therapy [1]. The reported incidence is variable, depending on the series reviewed. Garvey and colleagues reported their experience in 154 patients diagnosed with IE between 1968 and 1973 [2]. A presumed coronary embolism causing an AMI was reported in 7% of the patients in their series. Another case series from Russia reported a 10.6% incidence of septic embolism causing acute coronary ischemia in 11 out of 104 patients analyzed [3]. In the largest published series, acute coronary syndrome (ACS) was seen in 2.9% of the cases (14 out of 586 cases), and septic embolism as a cause of AMI was documented in only 0.51% (3 out of 586 cases) [4]. A more contemporary European multicenter prospective study corroborated these findings with a reported incidence of 0.52% (2 out of 384 cases) [5]. Embolic events were equally likely with native and prosthetic valves. Historically, mitral valve endocarditis was associated with a higher risk of embolic complications (overall incidence of 25% vs. 10% with aortic valve endocarditis) [2]. This belief was refuted by more recent data demonstrating that embolic events happen at comparable rates in aortic valve and mitral valve endocarditis [5].

We present a complicated case of an intravenous drug user with multiple previous admissions for both native and prosthetic valve endocarditis. In his most recent presentation, he developed an acute anterolateral ST-elevation myocardial infarction (STEMI). He underwent coronary angiography and primary revascularization of an occluded left anterior descending (LAD) coronary artery for persistent ST elevation and hemodynamic instability. He unfortunately died during the index hospitalization from mixed cardiogenic and septic shock. We also summarize our findings from a literature review on outcomes after percutaneous coronary intervention (PCI) compared to coronary artery bypass graft (CABG) surgery in managing this acute complication.

## Case presentation

**Figure 1:**
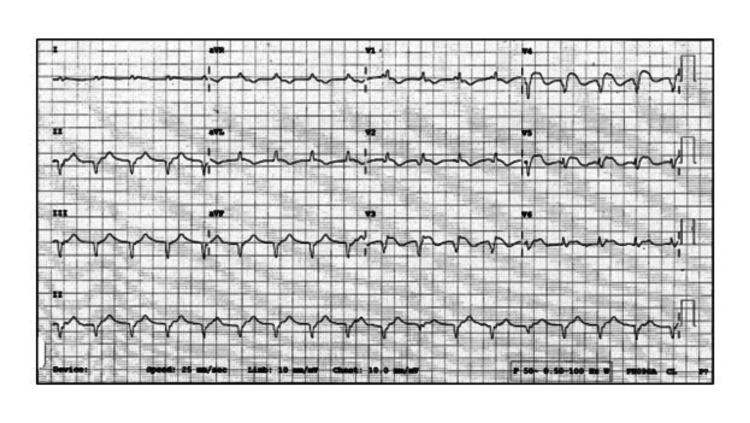
12-lead electrocardiogram obtained at the time of acute decompensation The tracing shows new ST-segment elevation in the anterior and lateral leads. The patient is in ventricular tachycardia with a right bundle branch block (RBBB) morphology, inferior axis, and atrioventricular dissociation.

A 51-year-old chronic intravenous drug user was admitted with a 2-day history of fever and rigors. In 2016, he underwent mitral valve replacement with a mechanical prosthesis for fungal mitral valve endocarditis. His hospital stay was complicated by multiple septic cerebral emboli and prolonged mechanical ventilation, culminating in a tracheostomy. He had no subsequent hospitalizations. On his current presentation, he was febrile (T = 39.8 °C) and hypotensive (BP = 84/54 mmHg), requiring parenteral norepinephrine. Precordial auscultation revealed an apical pansystolic murmur. His initial laboratory investigations showed leucocytosis with a leftward shift (WBC count 18.2x10^9^/L, polymorphonuclear leukocytes (PMN) 15.0x10^9^/L) and raised C-reactive protein at 182 mg/L. Renal function tests were normal. A bedside echocardiogram revealed a highly mobile, filamentous structure attached to the base of the non-coronary cup of the native aortic valve measuring 11 mm in its greatest dimension. The mass prolapsed into the LVOT during diastole. The left ventricular systolic function was normal at presentation. There were no visible vegetations on the mitral prosthesis. All three sets of blood cultures sent on presentation grew methicillin-sensitive *Staphylococcus aureus* within 24 hours of admission. Based on the antibiotic sensitivity report, treatment with parenteral clindamycin, amikacin, and cefazolin was initiated. On the fourth day, he became acutely hypoxic. His initial 12-lead electrocardiogram (ECG) demonstrated an evolving anterolateral STEMI (Figure 1). A repeat echocardiogram revealed severe hypokinesis of the anterior and anterolateral left ventricular (LV) walls with an estimated LV ejection fraction (LVEF) of 25%. The previously seen aortic valve vegetation was significantly smaller in size. He was sent to the catheterization laboratory for an urgent coronary angiogram.

**Figure 2:**
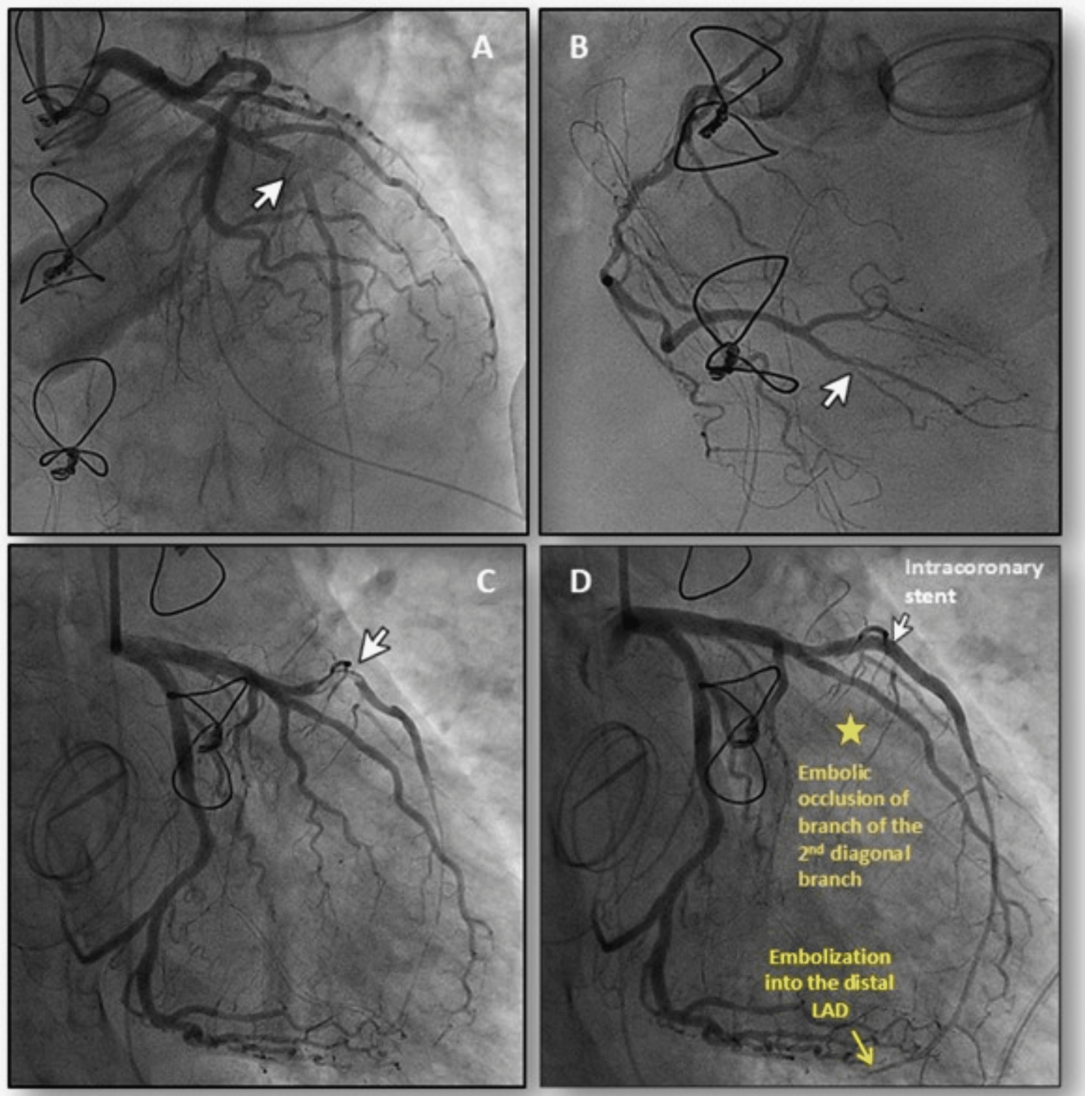
Representative still frames from the coronary angiogram 2A: cranial projection profiling the LAD along its course. There is a near-complete occlusion with a filling defect seen in the mid-LAD (white arrow: embolized vegetation). 2B: a left anterior oblique projection showing a long flow-limiting atherosclerotic plaque in the mid-segment of the RCA. A filling defect is seen in the body of the posterior interventricular branch of the distal RCA. 2C: a right anterior oblique projection of the left coronary artery. A filling defect is noted in the mid-LAD (white arrowhead). 2D: the same projection post-PCI showed a patent LAD with an abrupt cut-off in the terminal segment due to distal embolization. Furthermore, note the occluded (absent) diagonal branch indicated by a star. LAD: left anterior descending, PCI: percutaneous coronary intervention, RCA: right coronary artery

**Figure 3:**
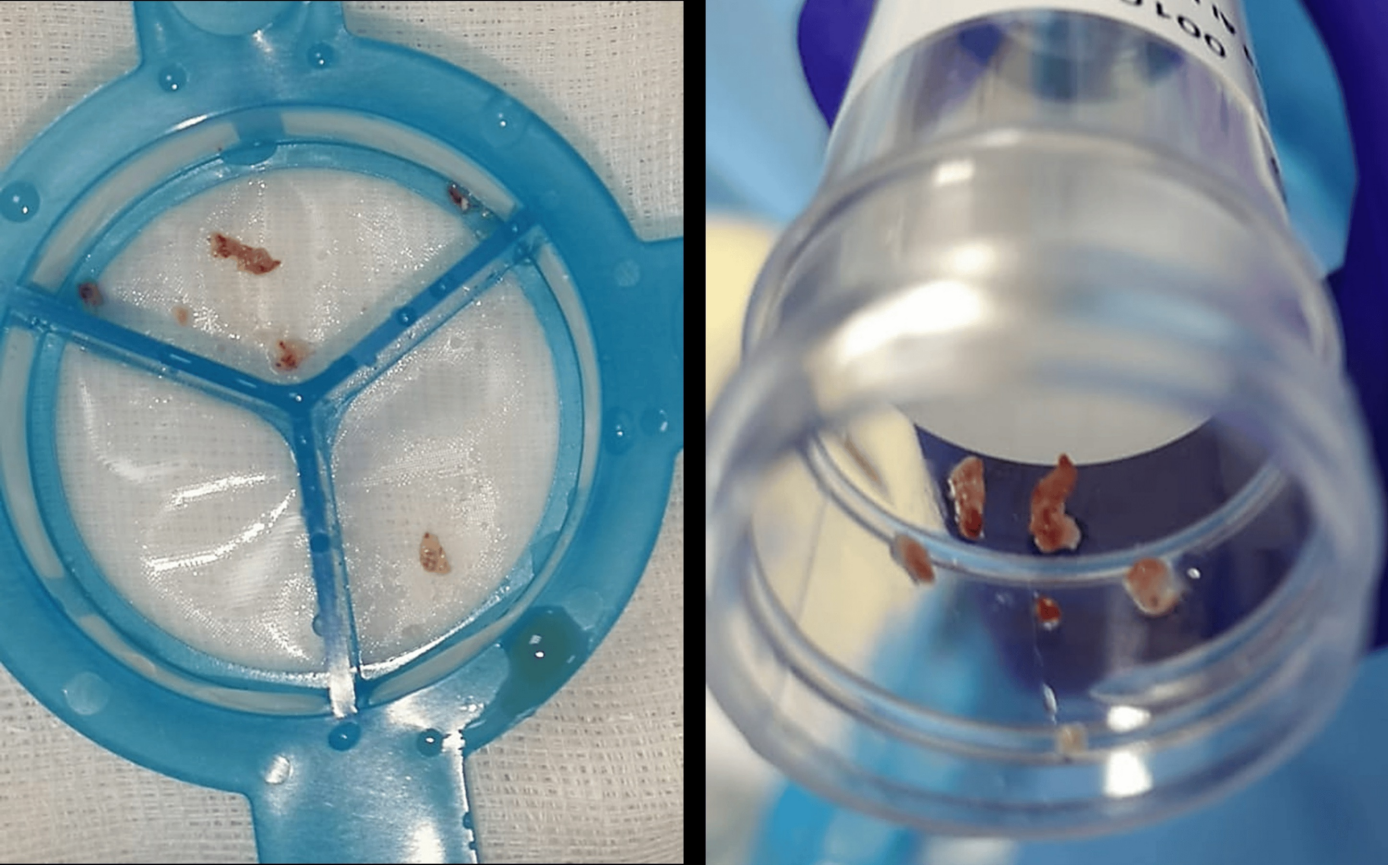
Aspirate recovered from the mid-LAD with an aspiration thrombectomy catheter LAD: left anterior descending

During the angiogram, LAD was found to be nearly occluded in the mid-segment, close to its bifurcation with a diagonal branch (Figure 2A). There was a small, non-flow-limiting embolus to the distal right coronary artery (RCA) (Figure 2B). Brisk thrombolysis in myocardial infarction (TIMI) III flow was seen in the RCA. After securing a coronary guidewire into the distal LAD, aspiration thrombectomy was performed, and retrieved fragments of while gelatinous material consistent with a fresh vegetation (Figure 3). Sluggish TIMI grade II flow was restored to the LAD, revealing further distal embolization that could not be aspirated. The large diagonal branch arising from the bifurcation was compromised and therefore treated with a 2.00x12 mm semi-compliant balloon, which restored TIMI III flow into the diagonal branch, but displaced the filling defect into the LAD with a visible dissection. This was sealed with a 3.00x28 mm drug-eluting stent (Figures 2C-2D).

The aspirates were sent for histopathological examination. It consisted of fragments of organized fibrinous deposits with enmeshed leucocytes, fragmented erythrocytes, and platelets. There were no bacterial or fungal elements identified. The final tissue culture did not grow any bacteria or fungi, and 16S rRNA sequencing did not reveal any bacterial DNA. This is likely due to several days of antibiotics received prior to the embolic event. 

Unfortunately, the patient remained in refractory mixed cardiogenic and septic shock, despite multiple inotropes. Given severe coagulopathy, thrombocytopenia, severe lactic acidosis, advanced critical state, and poor functional status at baseline due to previous hospitalization and cerebral embolism, the patient was not a candidate for mechanical circulatory support. He died two days after PCI.

## Discussion

Myocardial ischemia complicating infective endocarditis is a dreadful event. There is a lack of guidance in the reported literature on the best approach to management, especially given the unusually high risk for hemorrhagic complications, especially from concomitant cerebral embolisms and cerebral mycotic aneurysms. Acute coronary syndromes in this setting are associated with a high risk of heart failure, cardiogenic shock, and complete heart block. Manzano and colleagues reported a statistically significant doubling in mortality over a 12-month follow-up period (64.29% in ACS complicated IE vs. 32.33% in non-ACS complicated IE) [4]. Similarly, early mortality increases by more than two-fold. Albosta and colleagues demonstrated a higher in-hospital mortality in IE patients with MI (27.5% in IE complicated by MI vs. 10.8% in IE without an MI) [6].

Several mechanisms have been proposed for myocardial ischemia in IE. These include embolization of vegetations into the coronary arteries, intermittent occlusion of the coronary ostia by mobile and bulky vegetations, periannular extension of the infection with formation of an aortic root abscess compressing the epicardial coronary arteries, development of pseudoaneurysms, severe aortic regurgitation compromising diastolic coronary flow as well as coronary perfusion gradient by increasing LV diastolic filling pressure, and finally by imposing substantial demand-supply mismatch especially with pre-existing atherosclerotic coronary disease [4]. Manzano and colleagues identified extrinsic compression from periannular extension of the infection as the most common cause of myocardial ischemia in this context [4].

The overall reported incidence of coronary embolism in IE is variable but appears to be rather low. Fabri and colleagues described this complication in only 2 patients out of 629 (0.3%) patients with IE [7]. Whereas Manzano and colleagues confirmed coronary embolization in 14 out of 586 with IE (2.9%), half of those patients had an infected prosthetic valve, and more commonly with aortic valve endocarditis (12 out of 14 cases). *Staphylococcus aureus* was the most common organism associated with coronary embolism [4]. As shown by Nazir and colleagues, acute coronary occlusion manifesting as an acute STEMI was the first presentation in nearly 63% of ACS presentations in IE [8,9].

We reviewed the reported literature for cases of IE complicated by STEMI to understand the baseline clinical characteristics of patients affected with this complication and examine their outcomes after revascularization, either by PCI or CABG. The case reports included are summarized in Table 1. The observed mortality rate was intermediate between that reported by Manzano and Albosta (43.6% vs. 64.29% vs. 27.5%, respectively) [4,6]. The most commonly affected valve was the native aortic valve. The most commonly incriminated microorganism was *Staphylococcus aureus* (in 35.9% of cases), with embolized vegetations affecting the LAD most commonly (in 42.5% of cases). These findings are summarized in Table 2.

**Table 1:** Summary of relevant clinical characteristics and outcomes of all cases reported in the literature of IE complicated by STEMI AV: aortic valve, CABG: coronary artery bypass graft surgery, Cx: left circumflex artery, D1: first diagonal branch of the left anterior descending artery, ESBL: extended spectrum beta-lactamase producing bacteria, F: female, LAD: left anterior descending artery, M: male, MV: mitral valve, NA: not applicable, PCI: percutaneous coronary intervention, RCA: right coronary artery, IE: infective endocarditis, STEMI: ST-elevation myocardial infarction

No.	Author/year	Age	Sex	Valve	Type	Organism	Location of MI	IRA	Mode of Treatment	Outcome	Death	Embolism	Stroke
1	Singh et al. (2015) [10]	70	M	AV	Native	Staph. aureus	Anterior	RCA	CABG	Successful	Yes	Multiple	Yes*
2	Wojciuk et al. (2012) [11]	70	M	MV	Native	Β-hemolytic Step.	Non-anterior	Cx	PCI	Successful	No	Multiple	No
3	Hibbert et al. (2012) [12]	53	M	AV	Native	Staph. aureus	Anterior	LAD	PCI	Failed	Yes	None	No
4	DeKam et al. (2010) [13]	85	F	MV	Native	Staph. aureus	Non-anterior	D1	PCI	Successful	No	None	No
5	Murtaza et al. (2017) [1]	56	M	AV	Native	Enterococcus	Anterior	LAD	PCI	Successful	No	None	No
6	Bolton et al. (2020) [14]	53	F	AV	Native	Staph. aureus	Non-anterior	RCA	Hybrid^#^	Successful	No	Multiple	Yes*
7	Ghazzal et al. (2020) [15]	38	M	AV	Native	Unspeciated yeast	Anterior	LAD	CABG	Successful	No	None	No
8	Peddi et al. (2019) [16]	31	F	AV	Native	Staph. aureus	Ant + Inf	LAD	No intervention	NA	Yes	None	No
9	Maqsood et al. (2014) [17]	40	M	AV	Prosthetic	Candida albicans	Anterior	LAD	PCI	Successful	Yes	One	Yes*
10	Jenny et al. (2014) [18]	73	F	AV	Native	Staph. aureus	Anterior	RCA	No intervention	NA	Yes	None	No
11	Patel et al. (2011) [19]	59	F	AV	Prosthetic	Not specified	Anterior	LM	PCI	Successful	No	None	No
12	Baek et al. (2008) [20]	27	M	MV	Native	Strep. viridans	Anterior	LAD	CABG	Successful	No	None	No
13	Okai et al. (2009) [21]	53	M	MV	Native	Strep. sanguis	Anterior	LAD	CABG	Successful	No	Multiple	Yes**
14	Ahmed et al. (2023) [22]	62	F	AV	Native	Staph. aureus	Non-anterior	PDA	PCI	Failed	Yes	One	No
15	Doost et al. (2021) [23]	56	M	MV	Native	Staph. aureus	Non-anterior	Cx	Hybrid^#^	Successful	No	None	No
16	Mazzoni et al. (2021) [24]	52	M	AV	Prosthetic	Staph. Lugdunensis	Non-anterior	Cx	Hybrid^#^	Successful	No	None	No
17	Fujito et al. (2021) [25]	79	F	MV	Native	Strep. agalactiae	Anterior	LCA	No intervention	NA	Yes	One	Yes**
18	Joy et al. (2020) [26]	63	M	AV	Prosthetic	ESBL E. coli	Anterior	LCA	Hybrid^#^	Successful	No	None	No
19	Denegri et al. (2021) [27]	77	M	AV	Prosthetic	Staph. aureus	Anterior	LAD	PCI	Failed	Yes	One	No
20	Ben et al. (2019) [28]	71	M	TV	Native	Staph. aureus	Non-anterior	RCA	PCI	Successful	No	None	No
21	Calero-Núñez et al. (2018) [29]	67	M	AV+MV	Native	Strep. sanguinis	Non-anterior	Cx	Hybrid^#^	Successful	No	None	No
22	Calero-Núñez et al. (2018) [29]	59	M	AV+MV	Native	Staph. epidermidis	Non-anterior	Cx	Hybrid^#^	Successful	No	None	No
23	Zaman et al. (2019) [30]	35	F	AV+TV	Native	Enterococcus faecalis	Non-anterior	RCA	Hybrid^#^	Successful	No	None	No
24	Karaarslan et al. (2018) [31]	64	M	MV	Native	Staph. aureus	Non-anterior	RCA	No intervention	NA	Yes	One	No
25	Liu YH et al. (2018) [32]	38	F	MV	Native	Strep. cristatus	Anterior	LAD	CABG	Successful	No	None	Yes*
26	Campanile et al. (2018) [33]	82	F	AV	Prosthetic	Staph. aureus	Anterior	LCA	PCI	Failed	Yes	None	No
27	Joliat et al. (2017) [34]	68	F	MV	Native	Enterococcus faecium	Non-anterior	RCA	No intervention	NA	Yes	Multiple	Yes**
28	Usui et al. (2021) [35]	68	F	AV+MV	Native	Enterococcus faecalis	Anterior	LCA	CABG	Failed	Yes	One	Yes**
29	Kotkar et al. (2016) [36]	54	M	MV	Native	Actinobacillus urinae	Non-anterior	RCA	CABG	Successful	No	None	No
30	Caspar et al. (2014) [37]	43	M	MV	Native	Staph. aureus	Anterior	LAD	Surgery	Successful	No	Multiple	No
31	Regmi et al. (2017) [38]	69	M	MV	Native	Staph. aureus	Anterior	LAD	PCI	Failed	Yes	One	Yes**
32	Thompson et al. (2017) [39]	22	F	MV	Native	Not specified	Non-anterior	RCA	PCI	Failed	Yes	One	No
33	Winkler et al. (2016)[40]	67	M	MV	Native	Gemella	Anterior	LAD	No intervention	NA	Yes	None	No
34	Rischin et al. (2015) [41]	42	F	AV	Native	Not specified	Non-anterior	LAD	PCI	Successful	No	One	No
35	Seo et al. (2014) [42]	53	M	MV	Native	Aspergillus	Anterior	LAD	PCI	Successful	Yes	None	No
36	Gültekin et al. (2012) [43]	40	M	AV+MV	Prosthetic	Staph. Epidermidis	Anterior	LAD	No intervention	NA	Yes	None	No
37	Whitaker et al. (2011) [44]	71	M	AV	Prosthetic	Enterococcus faecalis	Anterior	LAD	Hybrid^#^	Successful	No	One	Yes*
38	Chen et al. (2007) [45]	39	M	MV	Native	Enterococcus faecalis	Non-anterior	RCA	No intervention	NA	No	None	No
39	Hussein et al. (2015) [46]	34	M	AV	Prosthetic	Aspergillus	Non-anterior	RCA	CABG	Successful	No	Multiple	No

**Table 2:** Summary of the clinical characteristics and mortality outcome in IE cases complicated by STEMI This table illustrates the general characteristics found in all 39 cases of IE complicated by a STEMI regardless of treatment. The difference in the number of vessels affected and valves and valve types affected is attributed to multiple valves or vessels involved simultaneously in a few of the cases studied. IE: infective endocarditis, LAD: left anterior descending artery, STEMI: ST-elevation myocardial infarction

Total IE cases reported	39 cases
Commonly affected vessel	LAD (17 out of 40, 42.5%)
Most common MI site	Anterior wall (23 out of 40, 56.1%)
Most affected valve	Aortic valve (22 out of 44, 50.0%)
Most affected valve type	Native valve (34 out of 44, 77.3%)
Most common causative organism	Staphylococcus aureus (14 out of 39, 35.9%)
Reported mortality	17 out of 39 cases (43.6%)

Given the rarity of the complication, there is a scarcity of evidence to guide recommendations on reperfusion strategies in MI complicating a septic coronary embolus. The choice of revascularization modality depends on the complexity of the coronary lesion, the severity of accompanying valvular lesions, the patient’s overall condition, and local expertise. The outcomes of patients after revascularization are not well represented in the literature. Table 3 summarizes all the literature reporting outcomes in patients undergoing PCI vs. CABG for STEMI complicating IE.

**Table 3:** Comparison of baseline characteristics and 30-day clinical outcomes in patients undergoing PCI vs. CABG for acute embolic myocardial infarction complicating infective endocarditis CABG: coronary artery bypass graft surgery, PCI: percutaneous coronary intervention

Characteristic	PCI [n=13]	CABG [n=4]
Age, mean + SD, yrs.	61.4 + 17.4	56.5 + 16.6
Male sex, n(%)	8 (61.5%)	3 (75%)
Pre-existing valve disease, n (%)	5 (38.5%)	1 (25%)
Prosthetic valves	3 (23.1%)	1 (25%)
Bicuspid aortic valve	2 (15.4%)	0
Rheumatic valve disease	0	0
Myxomatous valve degeneration	0	0
Affected valve		
Native, n(%)	9 (69.2%)	4 (80%)
Mitral	5 (38.5%)	2 (40%)
Aortic	3 (23.1%)	2 (40%)
Tricuspid	1 (7.7%)	0
Prosthetic	4 (30.8%)	1 (20%)
Mitral	0	0
Aortic	4 (30.8%)	1 (20%)
Tricuspid	0	0
Location of the MI, n(%)		
Anterior	8 (61.5%)	2 (50%)
Non-anterior	5 (28.5%)	2 (50%)
Causative microorganism, n(%)		
Staphylococcus spp.	7 (53.9%)	1 (25%)
Methicillin-sensitive Staph. aureus	6 (46.2%)	1 (25%)
Other	1 (7.7%)	0
Streptococcus spp.	1 (7.7%)	0
Beta-hemolytic strep.	1 (7.7%)	0
Other	0	0
Enterococcus	1 (7.7%)	1 (25%)
Yeast	1 (7.7%)	0
Other	3 (23.1%)	2 (50%)
30-day mortality, n(%)	8 (61.5%)	2 (50%)
Acute stroke, n(%)	2 (15.4%)	2 (50%)
Ischemic	1 (7.7%)	1 (25%)
Hemorrhagic	1 (7.7%)	1 (25%)
Revascularization failure	4	0
Acute stent thrombosis	1 (25%)	NA
Mycotic aneurysm	2 (50%)	0
Aneurysm rupture	1 (25%)	0
Coronary perforation	0	0
Surgical graft failure	NA	0
Number of systemic emboli, n(%)		
One embolic event	6 (46.2%)	3 (75%)
Multiple embolic events	6 (46.2%0	3 (75%)

As shown in Table 3, patients undergoing PCI are older than those undergoing CABG (61.5+17.4 vs. 56.5+16.6, respectively). Patients undergoing CABG are more likely to be males (75.0% vs. 61.5%). The incidence of stroke was markedly higher in patients undergoing CABG compared to those treated with PCI (50% vs. 15%). The reported 30-day mortality was high in both treatment groups, but interestingly, higher in the PCI-treated group compared to those who underwent CABG (61.5% vs. 50%). Lower reported mortality in CABG despite a higher stroke rate may reflect selection or reporting bias, as operable patients are preferentially referred for surgery, whereas PCI is often performed in patients deemed unsuitable for surgery. Other potential confounders include patient comorbidities, severity of infection, extent of valve involvement, and hemodynamic instability. A possible explanation for the increased mortality in the PCI-treated patients would be higher procedure-related complications. Manipulating diagnostic and guide catheters in the aortic sinuses while engaging the coronary ostia can potentially dislodge vegetations attached to the aortic valve and embolize them. Also, mechanical aspiration can result in distal embolization during catheter advancement. Stent implantation and balloon angioplasty in infected lesions increase the risk of stent infection and local mycotic aneurysm formation with concomitant risk of rupture and sudden death. No such complications were reported with CABG-treated patients in the cases extracted from the literature, which may account for the lower overall mortality rate in this group. Nonetheless, CABG was associated with different procedure-related risks, including risks related to cardiopulmonary bypass, longer operative time, intraoperative and postoperative bleeding, and post-surgical infection. Regardless of the proposed revascularization strategy, prompt targeted intravenous antibiotics and hemodynamic support remain essential. In the published reports, PCI was often selected for patients who were deemed unfit for surgery.

## Conclusions

In conclusion, an acute MI complicating IE is associated with substantial mortality and morbidity. There are no clear treatment recommendations. We report the case of a middle-aged intravenous drug user with recurrent native and prosthetic valve endocarditis who was admitted with methicillin-sensitive *Staphylococcus aureus* endocarditis of the native aortic valve. His course was complicated by an acute anterior STEMI from a septic embolus occluding the mid-LAD. Despite restoring TIMI II-III perfusion in the infarct-related artery, the patient succumbed to his disease and died from shock and multi-organ failure. Treatment decisions need to be tailored to the individual case and resources and expertise available at the treating center, taking into account patient factors, anatomical complexity, and institutional considerations, including surgical expertise and multidisciplinary decision-making.
